# A mathematical model to simulate the biological action of Infliximab on TNF-$\alpha$ in patients with Inflammatory Bowel Disease: the critical role of drug clearance

**DOI:** 10.1007/s11538-026-01603-9

**Published:** 2026-02-12

**Authors:** Ana M. Portillo, Ángel De Prado, Ana J. Soares

**Affiliations:** 1 Instituto de Investigación en Matemáticas, University of Valladolid, Paseo Belén, 47011 Valladolid, Spain; 2 Applied Mathematics, School of Industrial Engineering, University of Valladolid, Pso. Prado de la Magdalena 3-5, 47011 Valladolid, Spain; 3 Mucosal Immunology Laboratory, Instituto de Biomedicina y Genética Molecular (IBGM), University of Valladolid-CSIC, Calle Sanz y Forés, 3, 47003 Valladolid, Spain; 4 Servicio de Gastroenterología, Hospital Universitario Río Hortega, Calle de la Dulzaina, 2, 47012 Valladolid, Spain; 5 Centre of Mathematics, University of Minho, Campus of Gualtar, 4710-057 Braga, Portugal

**Keywords:** Inflammatory bowel disease, TNF-$\alpha$, Infliximab, Biologic therapy, Drug clearance, Mathematical modelling, 34A34, 65L05, 92C50, 92C45

## Abstract

Inflammatory bowel disease (IBD), including Crohn s disease (CD) and ulcerative colitis (UC), is characterized by chronic intestinal inflammation driven by elevated tumor necrosis factor-alpha (TNF-$\alpha$). Infliximab, an anti-TNF-$\alpha$ monoclonal antibody, is widely used in the treatment of inflammatory bowel disease but shows variable effectiveness due to interindividual pharmacokinetic diversity. We develop a low-dimensional mathematical model of ordinary differential equations to describe TNF-$\alpha$ dynamics, its interactions with receptors and infliximab, and the influence of drug clearance on treatment outcomes in CD and UC. This model is combined with a pharmacokinetic framework that enables the estimation of the infliximab clearance coefficient, which can then be used to guide dosage adjustments in the treatment. The model balances biological realism with analytical tractability, enabling rigorous mathematical analysis and numerical simulations. The parameters are adapted for CD and UC. The study investigates how drug clearance influences treatment efficacy, initially using constant clearance values and later incorporating values that vary with the level of inflammation. Simulations are performed across a range of clearance rates and dosing regimens, providing detailed insights into infliximab and TNF-$\alpha$ dynamics, as well as therapeutic drug monitoring parameters. Our results highlight the critical role of clearance and therapeutic drug monitoring in optimizing infliximab therapy. This approach offers valuable insights to support personalized treatment strategies in IBD.

## Introduction

Inflammatory bowel disease (IBD), including Crohn’s disease (CD) and ulcerative colitis (UC), is a chronic, relapsing-remitting condition characterized by immune dysregulation, alterations in gut microbiota, and compromised intestinal barrier function. Its incidence has been increasing globally over the past few decades, particularly in developed countries, reflecting the influence of environmental factors on disease pathogenesis (Ng et al. 2017). Although the precise etiology of IBD remains incompletely understood, it is widely accepted that it arises from a multifaceted interplay among genetic predisposition, environmental factors, microbial dysbiosis, and aberrant immune responses (Guan 2019).

Tumor Necrosis Factor-$\alpha$ (TNF-$\alpha$) is a pro-inflammatory cytokine that plays a central role in the pathogenesis of IBD. Elevated levels of TNF-$\alpha$ have been detected in the intestinal mucosa and serum of patients with active IBD, correlating with disease severity (Maeda et al. 1992; Owczarek et al. 2012). TNF-$\alpha$ contributes to the inflammatory cascade by promoting leukocyte recruitment, activating endothelial cells, and impairing the mucosal barrier function, which can lead to tissue damage (Liu et al. 2022). This understanding has led to the development of anti-TNF-$\alpha$ therapies, which have revolutionized the treatment landscape for IBD.

Infliximab, a chimeric monoclonal antibody targeting TNF-$\alpha$, was the first biologic agent approved for the treatment of CD and later UC and remains a cornerstone in the therapeutic arsenal. Clinical trials have demonstrated that infliximab is effective in inducing and maintaining remission, promoting mucosal healing, and reducing the need for corticosteroids and surgery (Rutgeerts et al. 2005; Hanauer et al. 2002). Nevertheless, primary non-response, loss of response over time, and potential adverse effects continue to challenge long-term disease control, underscoring the need for personalized treatment strategies and ongoing research (Fine et al. 2019; Kamal et al. 2024). To mitigate these risks and optimize treatment outcomes, therapeutic drug monitoring is employed to tailor dosing and assess drug levels and antibody formation.

Given the complexity of the immune mechanisms involved and the variability in patient response, quantitative approaches have become valuable tools to complement clinical and experimental research. In particular, mathematical models using differential equations are increasingly being applied to understand how the immune system works in IBD and to evaluate the pharmacodynamics of anti-inflammatory therapies. The model in (Park et al. 2020) employs a system of seventeen ordinary differential equations (ODEs) to describe the dynamic interactions between pro-inflammatory (Th1, Th17) and anti-inflammatory (Treg) T cell subsets, as well as cytokines such as TNF-$\alpha$, IL-6, and IL-10. The model incorporates the effect of anti-TNF therapy by simulating cytokine neutralization, revealing how immunoregulatory balances shift during treatment. The Dynamic Quantitative Systems Pharmacology model in (Rogers et al. 2021a, b) uses a system of thirty one ODEs and a large number of parameters to simulate biomarker dynamics (e.g., CRP, fecal calprotectin) and immune responses in Crohn s disease. It assesses patient responses to infliximab (anti-TNF) and ustekinumab (anti-IL-12/23).

The above mentioned high-dimensional models are valuable from a pharmacological perspective, especially for simulating complex biological processes and supporting drug development. However, their analytical tractability is limited due to the high dimensionality in terms of number of equations and parameters involved.

**Fig. 1:**
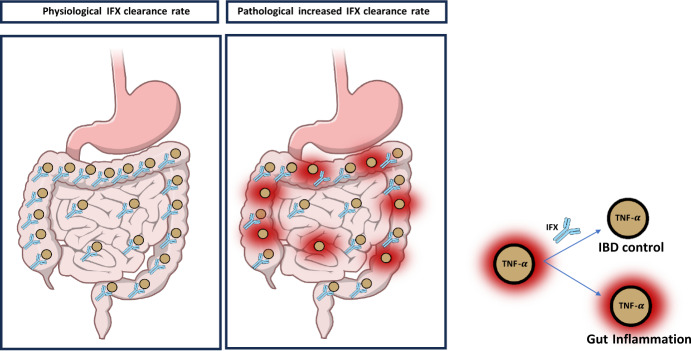
Impact of infliximab clearance on intestinal inflammation in IBD. The figure illustrates two contrasting states related to infliximab pharmacokinetics and their consequences in IBD. Left panel: In the physiological state, adequate drug exposure is maintained due to low clearance of infliximab, allowing sustained neutralization of tumor necrosis factor-alpha (TNF-$\alpha$). This promotes resolution of intestinal inflammation and restoration of mucosal integrity. Central panel: In the pathological state, accelerated clearance of infliximab results in subtherapeutic drug levels. Insufficient TNF-$\alpha$ neutralization leads to persistent cytokine activity, promoting chronic inflammation and mucosal damage in the gut. Right panel: When antibody-antigen complexes are formed in sufficient proportion IBD is controlled, otherwise Gut inflammation persists. Together, these contrasting scenarios highlight the critical role of maintaining therapeutic infliximab levels in achieving effective disease control in IBD.

In this work, we develop a mathematical model designed to balance biological realism with analytical simplicity, that still retains essential clinical features. Our mathematical model seeks to integrate clinical and pharmacokinetic factors to simulate TNF-$\alpha$ dynamics and infliximab action with only four equations and few parameters. The model enables both theoretical analysis, providing consistency and robustness of the approach, while also supporting practical numerical simulations based on clinical pharmacology. By quantitatively assessing the impact of the clearance rate infliximab, it aims to enhance precision in treatment optimization and predict long-term patient outcomes. Figure 1 displays the impact of infliximab clearance in two contrasting physiological states in IBD: the physiological state and the pathological state.

The dynamic interplay between TNF-$\alpha$ concentrations and infliximab levels represents a key determinant of therapeutic success. However, in current clinical practice, direct quantification of TNF-$\alpha$, either in serum or tissue, is not routinely performed, primarily due to technical variability, cost, and limited standardization across laboratories. Instead, therapeutic drug monitoring, which includes measurement of serum infliximab concentrations and anti-drug antibodies, is the established and clinically validated method for assessing treatment adequacy and guiding dose optimization in IBD management. Accordingly, our experiments were designed to reflect this real-world clinical framework, focusing on pharmacokinetic- pharmacodynamic relationships that can be directly correlated with measurable clinical parameters.

After this Introduction, the paper is organized as follows. In Section 2, we present our model, based on the ideas discussed in paper (Jit et al. 2005). The model describes TNF-$\alpha$ dynamics and its interactions with receptors and antibodies. In Section 3, we introduce a reduced version of the model with fewer equations, simplifying the mathematical analysis of the system. In Section 4, we establish key mathematical properties of the model, including existence, positivity, and boundedness of solutions, thus providing a rigorous framework supporting biological interpretations. Section 5 presents numerical simulations illustrating the model s dynamical behaviour under relevant biological scenarios and exploring different therapeutic conditions. Finally, Section 6 discusses the clinical implications of the model, highlighting how it captures key factors such as TNF-$\alpha$ dynamics, drug clearance, and therapeutic drug monitoring, and emphasizing the potential of the model to support personalized treatment strategies. The paper concludes with an appendix, including the MATLAB script used to reproduce one of the figures from the numerical simulations, to ennsure the reproducibility of the results.

## Mathematical model

The model developed here is based on the model (Jit et al. 2005) which represents TNF-$\alpha$ dynamics. As in (Jit et al. 2005), we assume that free TNF-$\alpha$ (with concentration *L*) can bind to cell-surface receptors (with total density $R_{tot}$) to form receptor- ligand complexes (with bound proportion *r* and unbound proportion $1- r$), or to antibodies (with concentration *A*) to form antibody -antigen complexes (with concentration *C*),

1 $$\begin{aligned} \begin{array}{ccc} & k_1& \\ & \longrightarrow & \\ L+(1-r)R_{tot} & & r R_{tot},\\ & \longleftarrow & \\ & k_{-1}& \end{array} \quad \quad \begin{array}{ccc} & k_a& \\ & \longrightarrow & \\ L+A & & C.\\ & \longleftarrow & \\ & k_{-a}& \end{array} \end{aligned}$$

The two reactions (1) are assumed to obey mass-action kinetics with association rates $k_1$ and $k_a$, and dissociation rates $k_{-1}$ and $k_{-a}$. Combining these reactions with the rate $\varepsilon$ of internalization of bound receptors and clearance rates $\delta _1$ for free TNF-$\alpha$, $\delta _a$ for free antibody and $\delta _c$ for antibody antigen complexes gives four coupled ordinary differential equations

2 $$\begin{aligned} \begin{aligned} \frac{dL}{dt}&=\omega _1-k_1R_{tot}(1-r)L+k_{-1}R_{tot}r-k_aLA+k_{-a}C-\delta _1L,\\ \frac{dr}{dt}&=k_1(1-r)L-k_{-1}r-\varepsilon r,\\ \frac{dA}{dt}&= \omega _a -k_a LA+k_{-a}C -\delta _a A,\\ \frac{dC}{dt}&= k_a LA-k_{-a}C -\delta _c C, \end{aligned} \end{aligned}$$

where $\omega _1$ represents the rate of production of TNF-$\alpha$ arising from some external stimulus and $\omega _a$ stands for the rate at which antibodies are introduced into the receptor compartment. In (Jit et al. 2005), the influence of autocrine signaling on TNF-$\alpha$ production is also taken into account. However, analysis reveals that under conditions of moderate autocrine response, the qualitative behavior of the system remains comparable to that observed in the absence of autocrine signaling. For the sake of simplicity the possible autocrine response is not considered in this work.

Given the prolonged duration of treatment benefits, it is necessary to modify the model to accurately reflect this sustained effect. It is proposed to change the constant value $\omega _1$ to a dynamic one depending on the variables of the model. To ensure that the system maintains the desired steady-state behavior, the production parameter $\omega _1$ in the equation (2) was replaced by an expression derived from the steady-state conditions.

For this purpose, we start by studying the equilibria of the system (2), solving the algebraic equations

3 $$\begin{aligned} &  \omega _1-k_1R_{tot}(1-r)L+k_{-1}R_{tot}r-k_aLA+k_{-a}C-\delta _1L=0, \end{aligned}$$

4 $$\begin{aligned} &  k_1(1-r)L-k_{-1}r-\varepsilon r=0, \end{aligned}$$

5 $$\begin{aligned} &  \omega _a -k_a LA+k_{-a}C -\delta _a A=0, \end{aligned}$$

6 $$\begin{aligned} &  k_a LA-k_{-a}C -\delta _c C=0. \end{aligned}$$

From (3), we obtain

$$ \omega _1 = R_{tot} \, \underline{ ( k_1(1-r)L - k_{-1}r \, ) } + \underline{k_aLA - k_{-a}C} + \delta _1L, $$

and from (4) and (6), the underlined terms are given by

$$ k_1(1-r)L-k_{-1}r=\varepsilon r, \qquad k_a LA-k_{-a}C =\delta _c C. $$

Using these relations in the above expression for $\omega _1$ yields

7 $$\begin{aligned} \omega _1=\varepsilon r R_{tot}+\delta _c C+\delta _1L. \end{aligned}$$

This reformulation guarantees that, in the absence of dynamic changes, the net input of *L* exactly compensates for the losses due to direct degradation $\delta _1L$, complex degradation $\delta _c C$ and dissociation or loss of the *r*-complex $\varepsilon r R_{tot}$. Therefore, substituting expression (7) into the first equation of system (2) for *L*, and simplifying $\delta _1L$, ensures biological consistency with the steady-state behavior and defines the new dynamics governing *L*. Then, in the new model

8 $$\begin{aligned} \begin{aligned} \frac{dL}{dt}&=\varepsilon r R_{tot}+\delta _c C-k_1R_{tot}(1-r)L+k_{-1}R_{tot}r-k_aLA+k_{-a}C,\\ \frac{dr}{dt}&=k_1(1-r)L-k_{-1}r-\varepsilon r,\\ \frac{dA}{dt}&= \omega _a(t) -k_a LA+k_{-a}C -\delta _a A,\\ \frac{dC}{dt}&= k_a LA-k_{-a}C -\delta _c C, \end{aligned} \end{aligned}$$

the production of the inflammatory response is dynamic. We consider initial data

9 $$\begin{aligned} L(0) = L_0>0 , \quad r(0) = r_0>0 , \quad A(0) = A_0\ge 0 , \quad C(0) = C_0\ge 0. \end{aligned}$$

We assume that $\omega _a(t)$, the source of the TNF-$\alpha$ inhibitor infliximab, is a continuous function, with value zero before the infusion, positive values during two hours of infliximab administration, and returning to zero at the end of infusion until the next infusion.

A comprehensive summary of the parameter values in the model (8), along with the corresponding references, is provided in Table 1.

**Table 1:** Estimates for the parameters used in the model (8) with corresponding sources from the literature.

Parameter	Meaning	Estimate	Reference
$k_1$	TNF-$\alpha$ receptor association rate	$1.7 \times 10^7$ $M^{-1}$ $s^{-1}$	(Grell et al. 1998)
$k_{-1}$	TNF-$\alpha$ receptor dissociation rate	$5.5 \times 10^{-4} s^{-1}$	(Grell et al. 1998)
$R_{tot}$	Maximum total receptor concentration	$1.5 \times 10^{-10} M$	(Imamura et al. 1987)
$\varepsilon$	Stimulated rate of endocytosis	$6 \times 10^{-4} s^{-1}$	(Mosselmans et al. 1988)
$\delta _1$	Clearance rate of TNF-$\alpha$	$10^{-5}s^{-1}$	(Smith et al. 1990)
$k_a$	Ligand Infliximab association rate	$10^6 M^{-1} s^{-1}$	(Northrup and Erickson 1992)
$k_{-a}$	Ligand Infliximab dissociation rate	$10^{-4} s^{-1}$	(Foote and Eisen 1995)
$\delta _c$	Clearance rate of ligand Infliximab complex	$8.5 \times 10^{-7} s^{-1}$	(Wagner et al. 1998)
$\delta _a$	Clearance rate of Infliximab	$[1.5,5]\times 10^{-6} s^{-1}$	(Klotz et al. 2007)
$\omega _a^*$	Infliximab infusion of 350*mg* for 2 hours	$3.3713 \times 10^{-10}M s^{-1}$	(Klotz et al. 2007)
$L^C_{eq}$	Equilibrium value of TNF-$\alpha$ in active CD patients	$2.07\times 10^{-11} M$	(Komatsu et al. 2001)
$L^{UC}_{eq}$	Equilibrium value of TNF-$\alpha$ in active UC patients	$1.40\times 10^{-11} M$	(Komatsu et al. 2001)

**Fig. 2:**
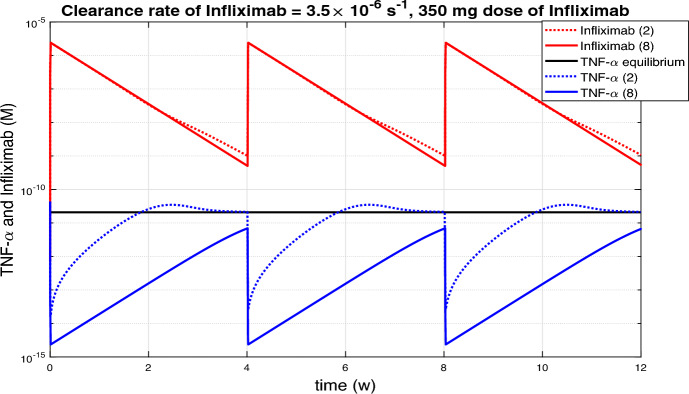
Time evolution of TNF-$\alpha$ and infliximab concentrations under a continuous 2-hour infusion of a $350\, mg$ dose of infliximab administered at weeks 0, 4, and 8 in CD patients, assuming a drug clearance rate of $\delta _a=3.5 \times 10^{-6} s^{-1}$. Results are shown with a dotted line for system (2) and a solid line for system (8). When the treatment was removed, TNF-$\alpha$ levels in system (2) increased rapidly and exceeded the equilibrium level by week 2, whereas in system (8), they remained below equilibrium for four weeks.

Figure 2 represents with a dotted line the solution of the system (2) and with a solid line the solution of the system (8) versus time, starting from $L_0= 4.35\times 10^{-11} M$, $r_0 = 0.0827$, $A_0=C_0=0$ so that the TNF-$\alpha$ equilibrium for active CD patients is $L_{eq}^C=2.07\times 10^{-11} M$ according to (Komatsu et al. 2001). An infliximab dose of $350\, mg$ administered via a 2-hour infusion is simulated at weeks 0, 4, 8 and $w_1 = 2.1315 \times 10^{-14} \,Ms^{-1}$. The evolution of TNF-$\alpha$ is shown in blue, and that of infliximab in red. Notice that when the treatment was removed, the TNF-$\alpha$ levels for system (2) grew very quickly above equilibrium $L_{eq}^C=2.07\times 10^{-11} M$, represented by the black horizontal line, and eventually tend to equilibrium before the next treatment. However, the TNF$\alpha$ concentration for system (2) remains below its equilibrium level for four weeks.

## Reduced system in *r*, *A* and *C*

In this section, we simplify system (8) by reducing it to an equivalent system with fewer equations, thereby facilitating the mathematical analysis developed in the next section. From (8), it is straightforward to obtain

10 $$\begin{aligned} \frac{dL}{dt} + R_{tot} \frac{dr}{dt} + \frac{dC}{dt} = 0 \end{aligned}$$

Integrating between 0 and *t*, we obtain

11 $$\begin{aligned} L(t) + R_{tot} \, r(t) + C(t) = L_0 + R_{tot} r_0 + C_0 \end{aligned}$$

This means that $L(t) + R_{tot} \, r(t) + C(t)$ remains constant in time. Introducing

$$ P_0 = L_0 + R_{tot} r_0 + C_0. $$

we can express one state variable in terms of the others. We choose to express *L* in terms of *r* and *C*, that is

12 $$\begin{aligned} L(t) = P_0 - R_{tot} \, r(t) - C(t), \end{aligned}$$

and obtain the following reduced system

13 $$\begin{aligned} \begin{aligned} \frac{dr}{dt}&= k_1 (1-r) L - k_{-1} r -\varepsilon r, \\ \frac{dA}{dt}&= \omega _a(t) - k_a LA + k_{-a}C - \delta _a A, \\ \frac{dC}{dt}&= k_a LA - k_{-a} C - \delta _c C, \end{aligned} \end{aligned}$$

where *L* is given by (12), that is

14 $$\begin{aligned} \begin{aligned} \frac{dr}{dt}&= k_1 (1-r) ( P_0 - R_{tot} \, r - C ) - k_{-1} r -\varepsilon r, \\ \frac{dA}{dt}&= \omega _a(t) - k_a A ( P_0 - R_{tot} \, r - C ) + k_{-a}C - \delta _a A, \\ \frac{dC}{dt}&= k_a A ( P_0 - R_{tot} \, r - C ) - k_{-a} C - \delta _c C. \end{aligned} \end{aligned}$$

When $\omega _a(t)=0$, the equilibria of the system (14) correspond to $A^*=0$, $C^*=0$, $r_1^*=(-b+\sqrt{b^2-4ac})/(2a)$ and $r_2^*=(-b-\sqrt{b^2-4ac})/(2a)$, for $a=k_1 R_{tot}$, $b=-k_1 (P_0 + R_{tot})-k_{-1}- \varepsilon$ and $c=k_1 P_0$. If $C_0=0$, then $L_1^*=L_0 + R_{tot} (r_0- r_1^*)$ and $L_2^*=L_0 + R_{tot} (r_0- r_2^*)$.

**Fig. 3:**
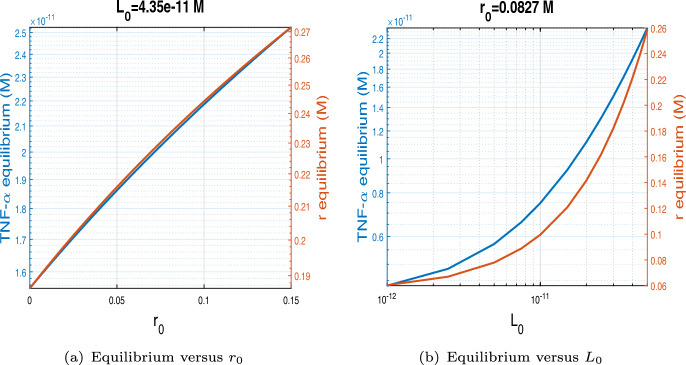
Biologically meaningful equilibria of the system (14) without treatment. (a) $L_0$ is fixed at $4.35 \times 10^{-11}$ while $r_0$ is treated as a continuous variable. (b) $r_0$ is held constant at 0.0827 while $L_0$ is considered over a continuous interval.

For the values of $k_1$, $k_{-1}$, $R_{tot}$ and $\varepsilon$ from Table 1 the equilibrium $L_1^*$ is negative while the other equilibrium $L_2^*$ is positive. This last one is the only one significant for our study. Such equilibrium typically represents a situation where no therapeutic antibodies are being administered, corresponding to a chronic inflammatory state, characterised by rather high TNF-$\alpha$ levels. Figure 3 depicts the biologically meaningful equilibrium of the system (14) without treatment. Figure 3 (a) displays TNF-$\alpha$ and *r* equilibrium versus $r_0$, for $L_0=4.35 \times 10^{-11}$ and Figure 3 (b) shows TNF-$\alpha$ and *r* equilibrium versus $L_0$, for $r_0=0.0827$.

## Mathematical properties of the model

In this section, we establish that the model is mathematically well-posed. Specifically, we analyse the existence, positivity, and boundedness of solutions, offering a mathematically robust framework that supports biologically meaningful interpretations in the context of CD and UC. This analysis also provides a solid basis for the numerical experiments developed in the next section.

### Theorem 1

(Local existence and positivity of the solution)

There exists $\bar{t}$ with $0 \!<\! \bar{t} \!<\! \infty$, such that system (8), with its initial data (9), admits a unique local positive solution defined on the time interval $[0,\bar{t}]$.

### Proof

We rewrite system (8) in vectorial form, obtaining

$$ \dot{U}(t) = F\big ( t,U(t) \big ) , $$

with $U=(U_1, \ldots , U_4)$ given by

$$ U(t)=\Big ( L(t),r(t),A(t),C(t)\Big ) \in \mathbb {R}^{4} $$

and $\displaystyle F: \mathbb {R_{+}} \times \mathbb {R}^4 \longrightarrow \mathbb {R}^4$ defined by

15 $$\begin{aligned} F\big ( t,U(t)\big ) \!=\! \begin{bmatrix} \displaystyle \varepsilon R_{tot} r(t) + \delta _c C(t) - k_1 R_{tot} (1-r(t) ) L(t)\\ + k_{-1} R_{tot} r(t) - k_a L(t) A(t) + k_{-a} C(t) \\ \displaystyle k_1 (1-r(t) ) L(t) - k_{-1} r(t) - \varepsilon r(t) \\ \displaystyle \omega _a(t) - k_a L(t) A(t) + k_{-a}C(t) - \delta _a A(t) \\ \displaystyle k_a L(t) A(t) - k_{-a} C(t) - \delta _c C(t) \end{bmatrix} \, . \end{aligned}$$

Then, the conclusion follows from the continuity of functions *F* and $\frac{\partial F}{\partial U}$ with respect to all components, by resorting to standard results from ODE theory given in Theorems 3.3 and 3.4 in book (Brauer and Nohel 1989).

Concerning the positivity, since $U_i(0) > 0$, for $i=1,\ldots ,4$, and

$$ U_i = 0 \; \Longrightarrow \; F_{i}(t,U(t)) \ge 0, \;\; \text{ for } \;\; i=1,\dots ,4\,, $$

then, by continuity arguments, see again book (Brauer and Nohel 1989), we have

$$ L(t)> 0, \;\; r(t)> 0, \;\; A(t)> 0 , \;\; C(t)> 0 , \;\; \text{ on } \;\; [0,\bar{t} \, ] \,. $$

$$\square $$

The above Theorem 1 states that there exists a non-negative solution of system (8) with initial conditions (9), defined on some, possibly small, time interval $[0,\bar{t}]$. The question now is to analyse whether this local solution can be extended to an arbitrarily large time interval. This will be addressed in Theorem 3, based on the uniform boundedness of the solution, which is is established in the following Theorem 2.

### Theorem 2

(Uniform boundedness of the solution) 

The solution of system (8) with their initial conditions (9) is uniformly bounded in any time interval $[0,\bar{t}]$ whenever it is defined, and satisfies the following conditions

16 $$\begin{aligned} r(t) \le 1, \quad A(t) \le \frac{\omega _a^*}{\delta } , \quad C(t) \le \frac{\omega _a^*}{\delta } , \quad L(t) \le P_0, \end{aligned}$$

where $$\delta = \min \{ \delta _a,\delta _c\}$$ and $$\displaystyle \omega _a^*=\max _{0\le t\le \bar{t}}\omega _a(t)$$.

### Proof

Let us consider the equivalent system formed by equations (12) and (14). We start with the first equation in (14) and write

$$ \frac{dr}{dt} \le k_1 (1-r) P_0 . $$

Integrating both sides between 0 and *t*, we obtain

$$ r(t) \le 1 + (r_0 - 1) e^{-k_1P_0 t}, $$

and therefore

$$ \lim \limits _{t\rightarrow +\infty } r(t) \le 1. $$

Now, we sum the last two equations in (14), resulting

$$ \frac{d}{dt} (A+C) \le \omega _a^* - \delta (A + C) . $$

Integrating between 0 and *t*, we obtain

$$ A(t) + C(t) \le (A_0 + C_0) e^{-\delta t} + \frac{\omega _a^*}{\delta } \Big (1 - e^{-\delta t} \Big ) , $$

where $\delta = \min \{ \delta _a,\delta _c\}$ and $\displaystyle \omega _a^*=\max _{0\le t\le \bar{t}}\omega _a(t)$. Therefore

$$ \lim \limits _{t\rightarrow +\infty } \Big [ A(t) + C(t) \Big ] \le \frac{\omega _a^*}{\delta } . $$

Since *A* and *C* are non-negative, the above condition indicates that both *A* and *C* are uniformly bounded. Finally, from equation (12) and from the positivity of functions *L* and *r*, *C*, the uniform boundedness of function *L* is straightforward. $\square$

### Theorem 3

(Global existence of the solution)

The unique solution of system (8) with their initial conditions (9) exists globally in time, that is, it is defined for all time $t \in [0,\infty [$.

### Proof

From Theorem 2, the unique solution is uniformly bounded. Therefore, using Theorem 3.6 from book (Brauer and Nohel 1989), we conclude that the solution exists and is unique on all time interval $[0,\infty [$. $\square$

## Numerical experiments

In this section, we carry out numerical simulations with the model developed and analysed in the previous sections, with the aim of illustrating its dynamical behaviour under biologically relevant scenarios and investigating its response to different therapeutic conditions. Although systems (8) and (14) are equivalent, the reduced system (14) is preferred for most of the qualitative analysis, given its lower dimension, while the full system (8) is more convenient for numerical simulations. We have numerically solved both systems using Matlab’s ode15s function, a variable-order, variable-step, implicit solver for stiff differential equations, based on backward differentiation formulas (BDFs), and verified that system (14) requires significantly more steps than system (8) under the same tolerance settings. This difference arises because, although both systems share the same solution, system (14) is stiffer, which forces the solver to take smaller step sizes.

In the appendix at the end of the paper, we include the MATLAB script illustrating how to determine the solution of system (8) using the ode15s solver.

The initial conditions used in our simulations were $A_0=0$, $C_0=0$, $r_0 = 0.0827$ and $L_0$ was chosen so that the equilibrium $L_2^*$ without drug coincides with the values in (Komatsu et al. 2001) (see Table 1). More specifically, $L_0= 4.35\times 10^{-11} M$ in CD and $L_0= 2.75\times 10^{-11} M$ in UC. We assume that $\omega _a(t)$, the source of the TNF-$\alpha$ inhibitor infliximab, takes the value $\omega _a^*$, a constant infusion for two hours, and then zero until the next infusion. The system (8) was numerically integrated using the Matlab’s solver ode15s with the parameters of Table 1.

### Effects of clearance rate of the inhibitor

**Fig. 4:**
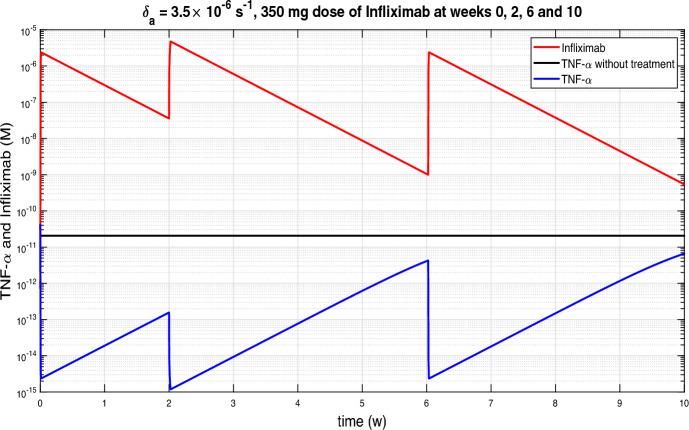
Continuous infusion for 2 hours of infliximab with 5 mg/kg dose, based on bodyweight of 70 kg, at week 0, 2, 6 and 10 in Crohn’s patients.

**Fig. 5:**
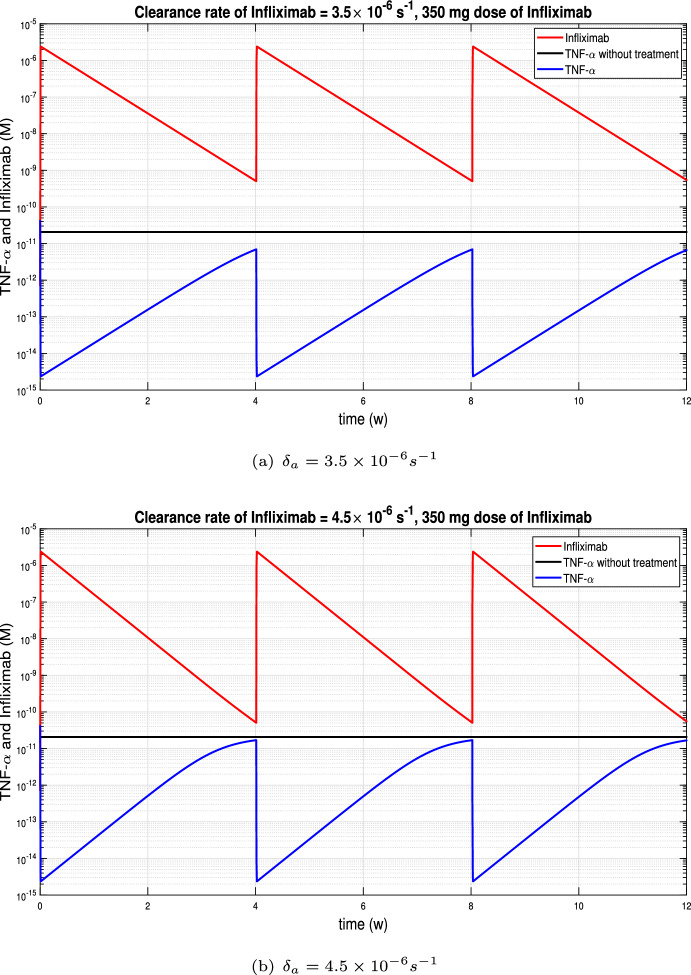
Continuous infusion for 2 hours of Infliximab with 350 mg dose at week 0, 4, 8 in Crohn’s patients with high rates of drug clearance. Peak TNF-$\alpha$ values are close to the TNF-$\alpha$ equilibrium $2.07\times 10^{-11} M$ without treatment.

In this subsection we consider periodic Infliximab’s doses of 350 mg by a 2-hour continuous infusion. In Figures 4 and 5(a) the same clearance rate of infliximab, $3.5 \times 10^{-6} s^{-1}$, was considered. On the one hand, in Figure 4 the infusions were at weeks 0, 2, 6 and 10 while in Figure 5(a) the infusions were every four weeks. It was observed that if the distance between infusions was shorter, then the TNF-$\alpha$ levels were lower, but if we move to a periodicity of 4 weeks, then the levels were equalised. In the rest of the subsection we will consider periodic infusions every 4 weeks and we will study the influence of the Infliximab clearance rate on TNF-$\alpha$ evolution.

**Fig. 6:**
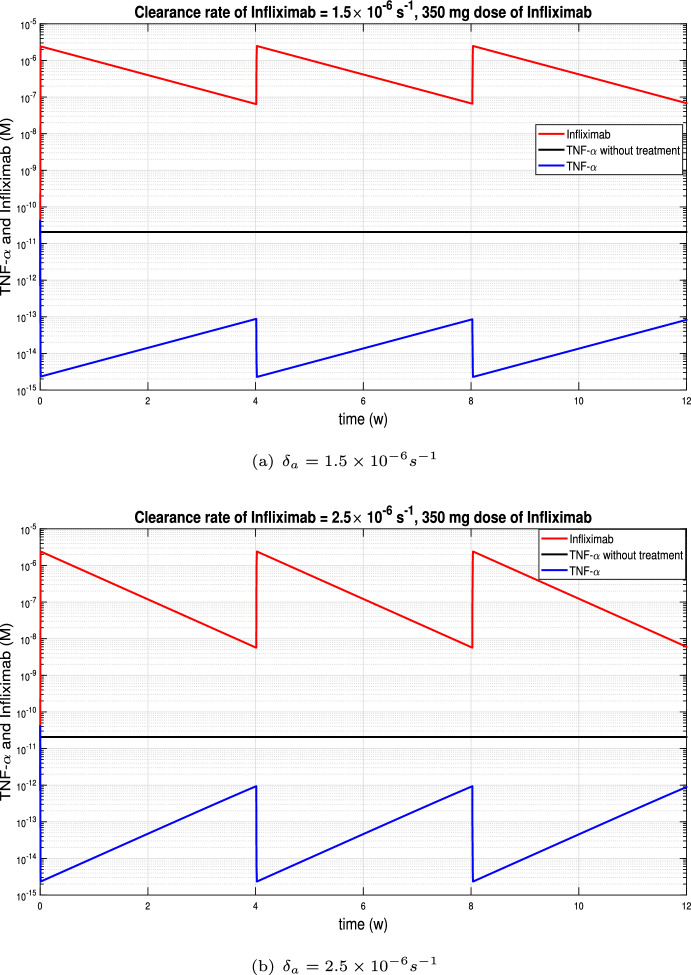
Continuous infusion for 2 hours of Infliximab with 350 mg dose at week 0, 4, 8 in Crohn’s patients with low rates of drug clearance. There is less distance between peaks and valleys. (a) TNF-$\alpha$ values are always less than $10^{-13} M$. (b) TNF-$\alpha$ values are always less than $10^{-12} M$.

Figure 5 displays the variation of TNF-$\alpha$ and Infliximab with high rates of drug clearance, while Figure 6 shows he corresponding evolution with low rates of drug clearance. Infliximab peaks coincide with TNF-$\alpha$ valleys, which occur after each infusion. Conversely, Infliximab valleys coincide with TNF-$\alpha$ peaks and occur just before the next infusion. Depending on the clearance rate of Infliximab, the distance between peaks and valleys varies, with greater distances observed for higher clearance rates. In Figure 5, where high rates of drug clearance are considered, peak TNF-$\alpha$ values are close to the TNF-$\alpha$ equilibrium $2.07\times 10^{-11} M$ without treatment. In contrast, in the Figure 6(a), the TNF-$\alpha$ values remain below $10^{-13} M$ throughout.

**Fig. 7:**
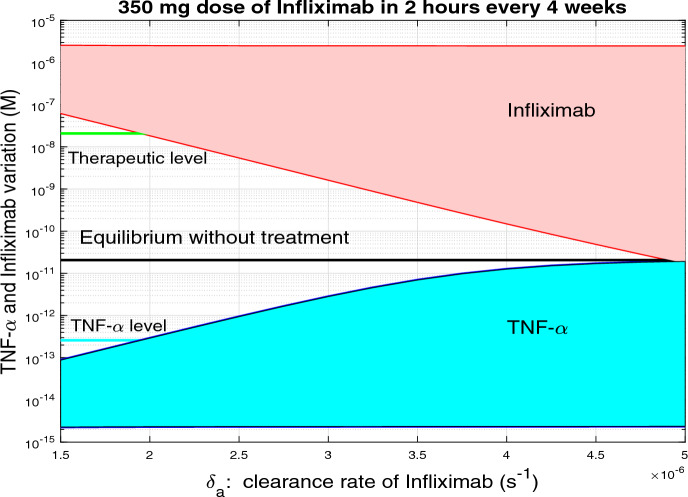
TNF-$\alpha$ and Infliximab variation as a function of the clearance rate of Infliximab, when a continuous infusion for 2 hours of Infliximab with 350 mg dose every 4 weeks is administered. The upper red line corresponds to the maximum values of Infliximab, while the lower red line represents the minimum values of Infliximab. Similarly, the upper blue line corresponds to the maximum TNF-$\alpha$, while the lower blue line represents the minimum TNF-$\alpha$ values. The black line depicts the equilibrium TNF-$\alpha$ value without treatment. The green line marks the value where the therapeutic range of Infliximab concentration begins (3*mg*/*L*). The cyan line indicates the TNF-$\alpha$ level ($2.6 \times 10^{-13}$ M), associated with the therapeutic drug level. When the clearance rate of Infliximab is less than $2 \times 10^{-6}$, the dose of 350 mg is within the therapeutic range, however as the clearance rate increases the minimum infliximab values are increasingly below the therapeutic range, so the drug dose should be increased. For clearance rate of Infliximab greater than $3.5 \times 10^{-6}$, the maximum TNF-$\alpha$ values approach the TNF-$\alpha$ equilibrium without treatment, which means that 350 mg dose has virtually no effect.

It seems clear that the effectiveness of treatment strongly depends on the clearance rate of the inhibitor. To study this influence in greater detail, we plot, in Figure 7, the variation of TNF-$\alpha$ and Infiximab concentrations with respect to Infliximab clearance rate. The top red line indicates the highest observed levels of Infliximab, while the bottom red line shows the lowest levels. Similarly, the top blue line represents the peak TNF-$\alpha$ concentration, and the bottom blue line indicates its minimum levels. The black line shows the baseline TNF-$\alpha$ concentration in the absence of any treatment. The green line marks the threshold at which Infliximab levels enter the therapeutic range. The cyan line indicates the TNF-$\alpha$ level corresponding to the minimum drug concentration required to achieve a therapeutic effect. The figure clearly shows that both levels are vertically aligned. Importantly, the TNF-$\alpha$ level $2.6 \times 10^{-13}$ M is inferred from the results produced by the model for Infliximab concentrations and corresponds to approximately $4.42 \times 10^{-6}$ mg/L. When the Infliximab clearance rate is below $2 \times 10^{-6}\, s^{-1}$, a dose of 350 mg falls within the therapeutic range. However, as the clearance rate increases, the minimum Infliximab levels drop below this range, indicating that a higher dose is needed to maintain therapeutic effectiveness. For clearance rates above $3.5 \times 10^{-6}\, s^{-1}$, the maximum TNF-$\alpha$ levels approach the untreated equilibrium level, suggesting that a 350 mg dose becomes largely ineffective.

### Infliximab clearance rate as a function of minimum Infliximab concentration

Increased intestinal permeability during active inflammation contributes to the fecal loss of anti-TNF-$\alpha$ agents, making their clearance dependent on the inflammatory burden (Brandse et al. 2015). To make the model more realistic, rather than keeping the Infliximab clearance coefficient constant, we will allow it to vary before each treatment, assuming that as inflammation decreases, the clearance rate decreases.

**Fig. 8:**
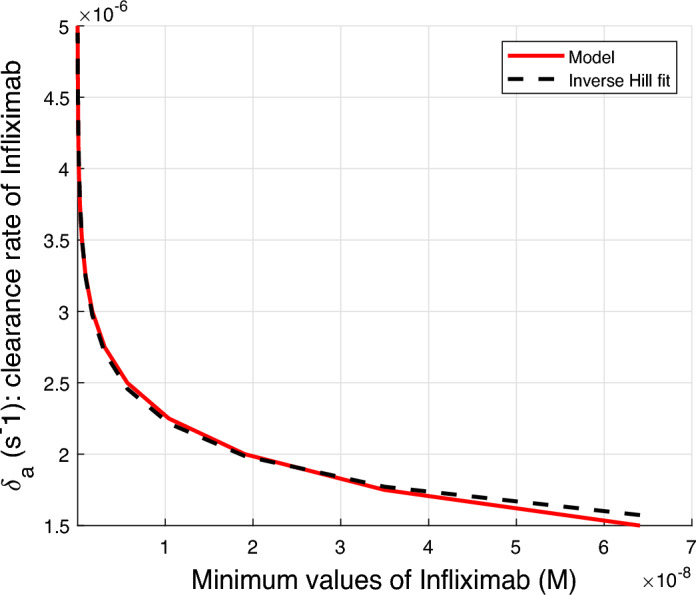
Clearance rate of Infliximab as a function of minimum values of Infliximab. The solid red line is constructed from the minimum values of Infliximab in Figure 7. The dashed black line represents the function $\delta _a(A_{min})=6.8939 \times 10^{-6} / \left( 1+(A_{min}/0.5809 \times 10^{-9})^{0.2590}\right)$, which corresponds to an inverted Hill model used to fit the data. This model effectively describes the inverse nonlinear relationship between the minimum Infliximab concentration and the associated clearance values: as trough concentrations increase, clearance decreases.

We use the inverted Hill model Weiss (1997); Hofmeyr and Cornish-Bowden (1997) to represent the relationship between the minimum Infliximab concentrations and the corresponding clearance values, as shown in Figure 7. The inverted Hill model was applied to fit the data, see Figure 8, using its standard Hill formulation given by

17 $$\begin{aligned} \delta _a(A_{min})=\displaystyle \frac{V}{1+(A_{min}/K)^{n}}, \end{aligned}$$

where $A_{min}$ represents the minimum (trough) concentration of Infliximab, represented in the lower red line of Figure 7; *V* is the maximum value of the clearance rate of infliximab, $\delta _a$, obtained when $A_{min}\rightarrow 0$; *K* is the value of $A_{min}$ at which $\delta _a$ reaches its half-maximal value, *i.e.* $\delta _a = V/2$ and *n* is the Hill coefficient controlling the steepness of the curve (Figure 8). In particular, larger values of *n* correspond to a more abrupt transition from high to low values of $\delta _a$ as $A_{min}$ increases. These three parameters are determined to provide the best fit to the data presented in the Figure 7. The estimated values obtained from the fitting process are $V = 6.8939 \times 10^{-6}$, $K = 0.5809 \times 10^{-9}$ and $n=0.2590$. In Figure 8, the dashed black line represents the inverse Hill fit obtained with equation (17), whereas the solid red line shows the dependence of the clearance rate $\delta _a$ on $A_{min}$, corresponding to the lower red line of Figure 7 evaluated from our model. It is clear that the inverted Hill model captures the inverse nonlinear trend observed in the data: as trough concentrations increase, clearance decreases.

A notable contribution of our study is the introduction of a variable drug clearance model that accounts for inflammation levels, capturing a clinically observed mechanism often neglected in existing models. It is reasonable to adjust the clearance rate $\delta _a$ before each new dose, since drug levels are typically measured prior to administration in clinical practice. We, therefore, consider a model in which the drug clearance coefficient may vary according to the Infliximab concentration before the next dose. Under this approach, the clearance is treated as constant over the four-week interval between treatments and is updated before each administration using the inverted Hill formula (17).

**Fig. 9:**
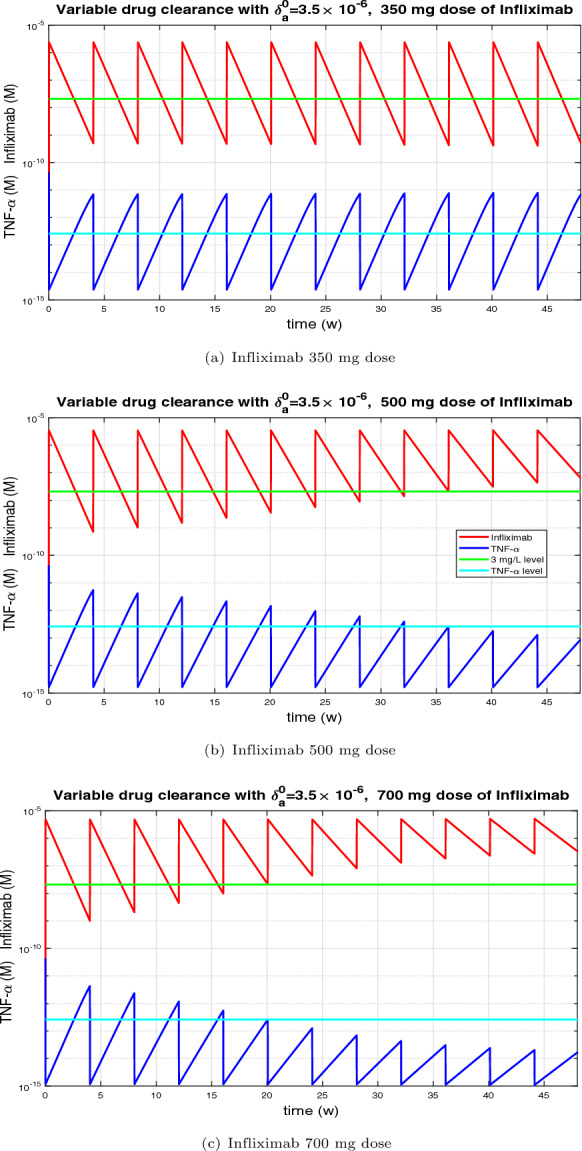
Simulation of one-year evolution of TNF-$\alpha$ and Infliximab in patients with Crohn’s disease, assuming Infliximab infusions were administered every 4 weeks. Initially, the drug clearance coefficient was set to $\delta _a^0=3.5 \times 10^{-6}\, s^{-1}$. Before each subsequent dose, it was updated based on the minimum drug concentration according to the expression $\delta _a(A_{min})=6.8939 \times 10^{-6} / \left( 1+(A_{min}/0.5809 \times 10^{-9})^{0.2590}\right) ,$ and was then held constant until the next infusion. (a) Dose of 350 mg: after one year, the therapeutic level was not achieved. (b) Dose of 500 mg: nine doses were required to reach the therapeutic level. (c) Dose of 700 mg: the therapeutic level was reached after six doses.

Figure 9 shows the evolution of TNF-$\alpha$ and Infliximab over one year of treatment in Cronh’s desease with this new model for three doses of Infliximab: 350 mg, 500 mg and 750 mg. The green line represents the value below which the treatment is considered to be in the operating range. An important characteristic that we can extract from Figure 9, is that with a dose of 350 mg after one year the treatment is not in range. A dose of 500 mg after one year would bring the treatment into range. Finally, with a dose of 750 mg after half a year the treatment would reach the range.

Similarly, we have analyzed the evolution of TNF-$\alpha$ and Infliximab over the course of one year of treatment for Ulcerative Colitis using the same drug clearance variable model and the same three doses of Infliximab. The resulting plots do not show substantial differences compared to those presented in Figure 9. Although the parameters are slightly different in UC compared to Crohn’s disease the evolution over a year is quite similar to that of Crohn’s disease. It is also noted that the dose of 350 mg does not reach the therapeutic range. The 500 mg dose takes less than a year to reach therapeutic levels, while the 750 mg dose takes half a year.

As known Ternant et al. (2013); Pouw et al. (2017), the clearance rate of Infliximab depends on individual patient characteristics. Being able to adjust the Infliximab dose based on an accurate estimation of each patient’s clearance rate would represent a major advantage that mathematical modelling could provide in supporting clinical decision-making in the management of Crohn’s disease and inflammatory bowel disorders.

**Table 2:** Number of Infliximab doses administered every 4 weeks required to reach the therapeutic level.

	Dose		
$\delta _a^0\, (s^{-1})$	350 mg	500 mg	700 mg
$2\times 10^{-6}$	2	1	1
$2.5\times 10^{-6}$	12	3	2
$3\times 10^{-6}$	-	9	6
$4\times 10^{-6}$	-	12	8
$4.5\times 10^{-6}$	-	-	10
$5\times 10^{-6}$	-	-	11

Table 2 shows the number of Infliximab doses administered every four weeks required to reach the therapeutic level, depending on the initial drug clearance $\delta _a^0$ and the administered dose in mg. The simulation was performed using the model with variable clearance based on Equation (17). According to Table 2, a dose of 350 mg is sufficient when the clearance coefficient is up to $2.5\times 10^{-6}\,s^{-1}$. For clearance values between $2.5\times 10^{-6}\,s^{-1}$ and $4\times 10^{-6} \,s^{-1}$ , the recommended dose increases to 500 mg. When the clearance exceeds $4\times 10^{-6}\,s^{-1}$, a dose of 700 mg is required.

Following the previous analysis, the proposed strategy involves administering a starting dose of 350 mg of Infliximab, followed by measurement of the Infliximab concentration after four weeks. The obtained data are then used in Equation (17) to estimate the drug clearance coefficient, which will subsequently guide any necessary dosage adjustments.

## Discussion

The current therapeutic arsenal for IBD offers a wide spectrum of interventions aimed at both acute flare control and long-term maintenance. Among these, biological agents have demonstrated superior efficacy over conventional therapies in rapidly resolving active inflammation. The European Crohn s and Colitis Organisation (ECCO) guidelines underscore the pivotal role of targeting TNF-$\alpha$ to rapidly control mucosal inflammation and prevent complications in moderate-to-severe CD and UC (Gordon et al. 2024; Raine et al. 2022).

Despite its clinical benefits, infliximab therapy is associated with significant challenges: approximately $10 - 30 \%$ of patients exhibit primary non-response and up to $50\%$ experience secondary loss of response over time (Chaparro et al. 2020). Additionally, adverse events, including infusion reactions and increased risk of opportunistic infections, require careful monitoring (Gordon et al. 2024). Conversely, discontinuation of anti-TNF therapy in patients in deep remission has been linked to higher relapse rates compared to continued treatment, supporting the importance of sustained TNF-$\alpha$ blockade (Gisbert et al. 2025).

Our mathematical model developed to simulate the pharmacodynamics of infliximab in IBD provides valuable insights into the intricate interplay between drug kinetics, TNF-$\alpha$ dynamics, and therapeutic outcomes. By integrating patient-specific variables such as baseline TNF-$\alpha$ levels, disease subtype and drug clearance rate, the model aims to deepen our understanding of infliximab s efficacy and guide optimized treatment strategies. One approach is to estimate the Infliximab clearance coefficient using Equation (17), which can then be employed to guide dosage adjustments throughout the course of treatment.

Elevated TNF-$\alpha$ concentrations are a hallmark of active IBD and contribute to its characteristic inflammatory milieu. By quantifying these differences, our model reinforces the rationale for targeting TNF-$\alpha$ and offers a framework for predicting individual responses to infliximab over time. While the model implicitly accounts for TNF-$\alpha$ dynamics within its mechanistic structure, it does not use an absolute TNF-$\alpha$ threshold for disease control, as such reference values are not currently standardized or clinically utilized. We consider that further studies incorporating quantitative TNF-$\alpha$ data could enhance the translational value of our model. Building on TNF-$\alpha$ dynamics, we propose the incorporation of baseline TNF-$\alpha$ quantification into the clinical assessment of candidates for anti-TNF therapy. Emerging evidence suggests that elevated TNF-$\alpha$ concentrations, either in serum or intestinal tissue, may predict a more robust therapeutic response to infliximab (Jessen et al. 2021; Cui et al. 2022). Measuring pre-treatment TNF-$\alpha$ levels could refine patient selection and support dose stratification. Integrating quantitative TNF-$\alpha$ assays into baseline evaluations may improve treatment algorithms and enhance response rates.

For patients who either fail anti-TNF therapy or exhibit biomarker profiles suggestive of poor suitability, such as low baseline TNF-$\alpha$ levels, alternative biologic and small-molecule therapies targeting distinct immune pathways have demonstrated. These include monoclonal antibodies such as ustekinumab (anti-IL-12/23) (Sands et al. 2019; Feagan et al. 2016), risankizumab (Louis et al. 2024; Peyrin-Biroulet et al. 2024), mirikizumab (D haens et al. 2023; Ferrante et al. 2024) (anti-IL-23), and vedolizumab (Feagan et al. 2013; Sandborn et al. 2013) (anti-$\alpha 4\beta 7$ integrin), as well as Janus kinase inhibitors (JAKi) like tofacitinib (Sandborn et al. 2017; Panés et al. 2017) and upadacitinib (Danese et al. 2022; Loftus Jr et al. 2023), which modulate intracellular cytokine signaling. Taken together, these complementary strategies, encompassing biomarker, based selection and alternative therapies, enables treatment decisions tailored to each patient s immunological profile.

Our model also identifies infliximab clearance as a key determinant of therapeutic efficacy. Evidence from IBD and other immune-mediated diseases, such as rheumatoid arthritis and ankylosing spondylitis, indicates that factors including body weight, anti-drug antibodies, serum albumin, glucose levels, and C-reactive protein (CRP) can significantly impact drug clearance (Eser et al. 2021; Brandse et al. 2017). These factors independently affect infliximab pharmacokinetics, and variations in clearance may lead to subtherapeutic exposure and diminished efficacy. Monitoring these parameters is therefore essential to guide personalized dose adjustments.

Therapeutic drug monitoring (TDM) emerges as a cornerstone in the management of infliximab therapy (Chaparro et al. 2020; Sánchez-Hernández et al. 2020). By measuring serum drug levels and anti-drug antibodies, clinicians can assess treatment effectiveness, identify potential loss of response, and guide adjustments in dosing or therapeutic approach. The integration of TDM data into the model enhances its predictive capacity and aligns with current clinical practices aimed at optimizing biologic therapy in IBD.

In conclusion, the proposed mathematical model serves as a valuable tool for simulating infliximab dynamics in IBD, offering a comprehensive approach that encompasses TNF-$\alpha$ levels, disease-specific pharmacokinetics, and therapeutic drug monitoring parameters. By providing a nuanced understanding of these factors, the model holds promise for improving patient outcomes through personalized treatment strategies.

## Data Availability

‘Not applicable’

## Appendix

The MATLAB script used to determine the solution of system (8) with the ode15s solver and to generate Figure 5(a) is included here to help readers reproduce the results.

## Declarations

### Materials availability

The datasets used in this study were obtained from previously published articles and are publicly available. ‘Not applicable’

### Code availability

The code is easily reproducible. The appendix includes, as an example, the MATLAB code used to generate Figure 5(a).
